# Nurse effects on measurement error in household biosocial surveys

**DOI:** 10.1186/s12874-020-00922-2

**Published:** 2020-02-27

**Authors:** Alexandru Cernat, Joseph W. Sakshaug

**Affiliations:** 1 University of Manchester, Humanities Bridgford Street, Manchester, M13 9PL UK; 2 Ludwig Maximilian University of Munich, Munich, Germany; 3 University of Mannheim, Mannheim, Germany; 4 Institute for Employment Research, Regensburger Strasse 104, 90478 Nuremberg, Germany

**Keywords:** Biosocial surveys, Measurement error, Nurse effects, Anthropometric measures

## Abstract

**Background:**

Biosocial survey data are in high demand, yet little is known about the measurement quality of health measures collected by nurses in respondents’ homes. Our objective was to analyze the degree to which nurses influence measurement in anthropometric and physical performance indicators collected from respondents in two nationally-representative UK biosocial surveys.

**Methods:**

The English Longitudinal Survey of Ageing and the UK Household Longitudinal Study – Understanding Society were used to analyze fourteen anthropometric and physical performance measures covering weight, height, pulse, grip strength, and lung capacity. Cross-classified multilevel models were used to estimate “nurse effects” on measurement error.

**Results:**

Overall, there is a medium effect of nurses on measurement. Across all measures collected in both studies, nurses explain around 13% of all measurement variation. Variation in specific measures range between approximately 2 and 25%. Grip strength and lung capacity are more heavily influenced by nurses than are height, weight, and pulse. Lastly, nurse characteristics explain only a very small proportion of nurse measurement variation.

**Conclusion:**

Objective health measures collected by nurses in household biosocial surveys are susceptible to non-trivial amounts of measurement variation. Nurse ID numbers should be regularly included in biosocial data releases to allow researchers to account for this unnecessary source of variation. Further, researchers are advised to conduct sensitivity analyses using control variables that account for nurse variation to confirm whether their substantive findings are influenced by nurse measurement effects.

## Background

The collection of objective health measures (or “biomeasures”) in population-based social surveys has become increasingly prevalent over the years [1]. At present, numerous surveys collect anthropometric measures (e.g. height, weight, waist and hip circumference, blood pressure), physical performance measures (e.g. grip strength, lung function), and biological specimens (e.g. blood, urine) alongside traditional survey measures [2–5]. These so-called “biosocial surveys” provide researchers with the means to enhance their understanding of the complex interrelationships between the social environment and health outcomes in the population [6–8].

There are at least three models of collecting biomeasures in social surveys. One model consists of a center-based assessment, where respondents present themselves at a medical facility (hospital, clinic, health center) [9,10]. This model allows for a wide range of biomeasures to be collected with sophisticated equipment administered by a licensed medical professional. However, this is the costliest model and impractical for older, more vulnerable populations [11]. A less expensive model is to train lay interviewers to administer the biomeasures inside respondents’ homes [12–16]. This model conveniently allows the administration of both the traditional interview and collection of biomeasures in a single visit. However, the range of biomeasures that can be collected through this model is more limited as some countries require certain specimens (e.g. whole blood) to be collected by a licensed medical professional. A compromise on the center-based and lay interviewer models is to send licensed nurses to respondents’ homes at some point after the traditional interview takes place [3, 17]. This model allows for the collection of a broader range of biomeasures compared to the lay interviewer model and at a significantly lower cost compared to the centre-based model.

Any model that deploys actors to collect biomeasures in nonclinical and nonstandardized settings poses challenges that can potentially affect the quality of the collected measures [18]. Quality assessments of biomeasures collected by lay interviewers have been documented [4, 15, 19, 20], but quality assessments for nurses who are deployed to respondents’ homes in social surveys are still lacking. A large body of work indicates that nurses are prone to committing measurement errors in clinical settings [21–29]. Imprecision of measurement (or between-observer variation) is the most commonly cited source of measurement error in nurse-led assessments [28, 29]. Biomeasures found to be susceptible to nurse measurement error, include height, weight, and waist and hip circumference [28], blood pressure [21, 22, 25], and skin fold measurements [28]. Multiple factors have been suggested as possible causes of nurse measurement error for these anthropometric measures, such as incorrect cuff size, variation in training, digit rounding, inadequate knowledge of appropriate measurement techniques, and the use of multiple nurses [24, 28, 30–35].

Suggested guidelines for minimizing nurse measurement error include adequate training, ongoing quality control, a controlled working environment free of impediments, double measurement of a subsample to assess the extent of imprecision, and minimizing the number of nurses used within a given study [28, 34,36–38]. However, these guidelines are incongruent with the practical realities of most large-scale biosocial surveys. First, freelance nurses are decentralized and geographically dispersed which makes standardized training and ongoing oversight of their technique rarely feasible. Second, the working environment (i.e. respondents’ homes) can vary considerably with respect to space, lighting, and other factors that may influence measurement. Third, double measurement is impractical as it places undue burden on respondents and may harm participation rates in follow-up waves of a longitudinal study [39]. Lastly, many nurses are needed to cover large geographical areas common in nationally-representative biosocial surveys. Deploying a large number of nurses increases the possible magnitude of measurement error, even when only small differences in technique occur between nurses over time [28].

Given the high demand for biosocial survey data, it is important to understand their impact on the measures collected. Only after that can the quality of these measurements be determined. While some biomeasures are relatively simple to administer and require modest amounts of training (e.g. anthropometric measures), there are others which are more complex and require more extensive training (e.g. physical performance measures). Assessing the degree of nurse measurement error across multiple biomeasures with different administration difficulties is therefore needed to determine where larger measurement errors occur and where improvements in measurement technique are most needed.

In this article, we examine the extent of nurse effects in two longitudinal household biosocial surveys: the English Longitudinal Study of Ageing (ELSA) and Understanding Society – the UK Household Longitudinal Study (US). Nurse effects are defined as variability in the measurements at the nurse level. This form of nurse measurement error introduces non-zero correlations among the measurements collected by a nurse, which can inflate the variance of descriptive estimates. Using a cross-classified multilevel model that separates nurse and area effects, we estimate nurse effects for a host of anthropometric and physical performance measures collected over multiple waves of each study. Lastly, we make use of background data on the nurses themselves to determine whether their age and level of experience are factors that explain nurse measurement error and should be accounted for in analyses of biosocial survey data.

In short, the following three research questions are addressed:

1. To what extent do nurses contribute to measurement error in biomeasure collection?
2. Are nurse measurement error effects consistent across surveys and over time?
3. Do nurse characteristics explain a significant amount of nurse measurement error?

## Methods

### Data sources

The UK Household Longitudinal Study (UKHLS) is a panel survey representative of the UK population [40]. It initially started in 1992 under the name British Household Panel Survey (BHPS). In 2009 a new version of the survey was implemented with a sample size of 40,000 households in the UK under the name Understanding Society (US). In wave 2 of the Understanding Society (USW2), a random 80% of the sample was selected for a nurse visit. Trained nurses visited respondents in their homes around 6 months after the main interview. The nurse visit collected data on height, weight, pulse, grip strength and lung capacity after receiving verbal consent. Additionally, after receiving written consent nurses also collected whole blood from the vein, but this component is not part of the present investigation. In wave 3 of Understanding Society (or wave 19 of the BHPS; BHPSW19), all eligible BHPS members were selected for a nurse visit. Similar procedures were followed as in the previous wave.

In US wave 1 the individual response rate was 81.8% while in waves 2 and 3 they were 59.4 and 61.3%, respectively [41]. Response rates for the nurse visit (among eligible individuals) were 58.6% for USW2 and 57% for BHPSW19 [42].

The English Longitudinal Study of Ageing (ELSA) is a longitudinal study that collects information every 2 years from a representative sample of residents in England who are 50 years of age and older [17]. The sample is based on respondents from the Health Survey for England. In ELSA waves 2 (ELSAW2), 4 (ELSAW4), and 6 (ELSAW6), nurses visited eligible respondents and collected the same anthropometric and physical performance measures as in the US/BHPS studies, as well as blood from the vein.

For ELSA waves 2, 4 and 6 the individual response rates were 82, 71, and 76%, respectively [43–46], while response rates for the nurse visits were 87.3, 85.7 and 84.3%, respectively, among eligible individuals [47].

### Outcomes

To investigate the impact of nurse effects on measurement error we consider five different outcome measures: height, weight, pulse, grip strength, and lung capacity. These cover the typical anthropometric and physical performance measures collected by nurses. Some of these measures are administered multiple times. For example, pulse is measured three times, grip strength is measured three times for each hand, and lung capacity is measured three times based on the equipment used. Table 1 shows all of the measurements collected. In total, fourteen individual measurements were taken [42,48]. In the forthcoming analysis, each individual measurement is analyzed separately to evaluate whether nurses have a differential impact on the full range of measurements they collect.

**Table 1:** Measures collected and analyzed in the US/BHPS and ELSA surveys

Type	Measure	Units	Number of measurements	Name in data
Anthropometric measures	Height	cm	1	height
	Weight	kg	1	weight
	Pulse	Beats per minute	3	pulse
Physical performance	Grip strength	Individual readings for non-dominant hand in kg	3	mmgsn
		Individual readings for dominant hand in kg	3	mmgsd
	Lung capacity	The amount of air that can be blown out in one second, measured in liters	1	htfev
		The speed of air moving out of lungs at the beginning of expiration, measured in liters per second	1	htpev
		The total amount of air that can be forcibly blown out after a full inhalation, measured in liters	1	htfvc

To investigate the influence of nurses on measurement we adopt similar procedures to those used in the interviewer effects literature [49]. The main challenge in this research is separating the effect of the nurse from other possible confounders, especially area effects and respondent characteristics. In the absence of randomized allocation of nurses to respondents, a statistical approach is needed to control for these confounders. To separate nurse and area effects, a cross-classified multilevel model is used with random effects for nurses and areas [50]. Here, areas are defined as Lower Super Output Areas that represent areas of approximately 1500 households.

Respondent characteristics are introduced as control variables. The control variables used for both surveys are: sex, age, having a partner, owning the house, education, overall health, if they have a long-term illness, if they live in London and if they live in the north of UK. Additional variables are included for the models based on the US data: if living in an urban area, household size, and interest in politics. In ELSA, a variable was included on whether the respondent lives alone.

### Statistical analyses

The cross-classified multilevel model is defined as:

$$ {Y}_{i\left(j,k\right)}={\gamma}_0+\sum {\gamma}_h{x}_{i\left(j,k\right)}+{U}_{0j}+{U}_{0k}+{\varepsilon}_i $$

where *Y*, the dependent variable, varies by individual (*i*), area (*j*), and nurse (*k*). This model is explained by an intercept (*γ*_0_) term and *h* control variables with fixed effects (*γ*_*h*_). The random effects for area (*U*_0*j*_) and nurse (*U*_0*k*_) are cross-classified. Lastly, *ε*_*i*_ represents the residual, or unexplained variance.

In the context of the present research, *U*_0*k*_ represents nurse effects. It represents the amount of variation that is explained by nurses after controlling for respondent characteristics and area effects. If nurses have no impact on the collection of a biomeasure this should be close to zero. This quantity will answer the first research question. To answer the second question, we inspect how this coefficient varies by the survey, wave of data collection, and biomeasure.

To answer the third research question, the model is expanded to include nurse control variables. Two nurse-level variables were collected in both surveys: nurse age and nurse experience. Comparing the estimate of *U*_0*k*_ in the first model and in the expanded model will inform whether these two characteristics explain the nurse measurement effects and whether they should be routinely collected in biosocial surveys.

R 3.5.2 was used for data cleaning and running the models. The models were estimated using the rstanrm package, an interface that facilitates the estimation of multilevel models using the Monte Carlo Markov Chain procedures from Stan. Estimation was implemented using four chains with 2000 iterations out of which the last 1000 were used for sampling. Weakly informative prior distributions were used: normal distribution for the intercept and slope and an exponential distribution for the residual.^1^

Missing data was handled using listwise deletion. Over all five samples there was, on average, about 4.6% missing cases on the independent variables and 2.5% on the dependent variables. Over all the samples, an average of 8798 respondents, 4735 areas, and 116 nurses are used per survey. The full sample and descriptive statistics can be found in [Additional file 1.docx].

## Results

### Magnitude of nurse effects

To answer the first research question, a cross-classified model was estimated for each of the fourteen measurement outcomes of interest in each of the five waves of data collection. To facilitate the interpretation of the results, the nurse random effects are represented as Intraclass Correlation Coefficients (ICCs). These can be interpreted as the proportion of variation that is associated with each level of the data, in our case: nurses, areas, and residual variance after controlling for respondent characteristics.

**Fig. 1:**
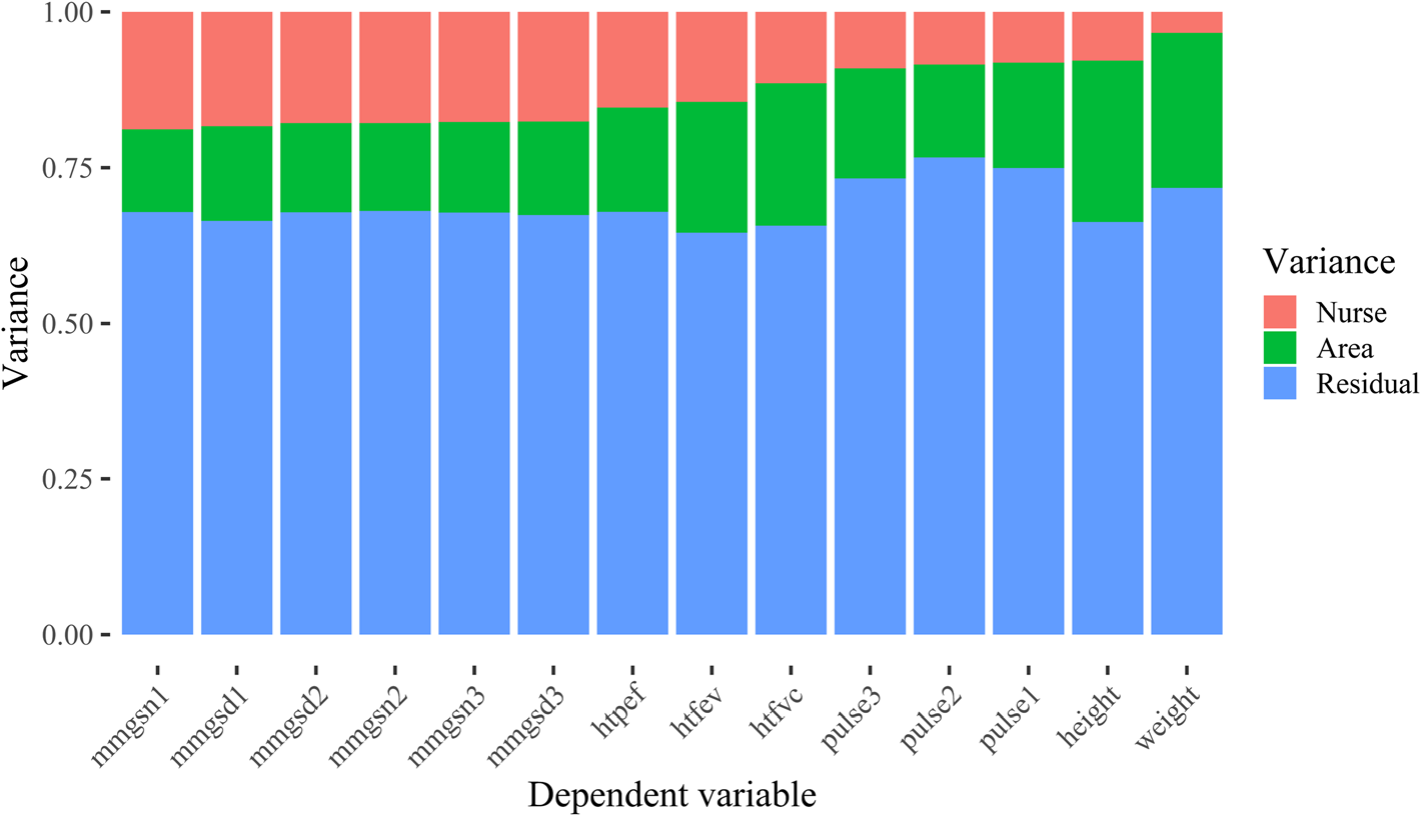
Variance decomposition for nurse visit measures after controlling for respondent characteristics

Across all biomeasures collected in all surveys and waves, the average nurse effect on measurement is 13%, which is considered to be a medium-sized effect. This quantity varies by biomeasure with a minimum of 2% to a maximum of 27%. Figure 1 shows how the nurse effects vary by type of measure collected. The largest nurse effects are for the physical performance measures: grip strength and lung capacity, while the smallest effects are for the anthropometric measures: pulse, height and weight. There also appears to be some variation within each measure depending on the order in which the measurements were collected. For example, the largest nurse effect for grip strength occurs at the first measurement, whereas the largest nurse effect for pulse is observed at the third measurement, although the differences are small.

### Nurse effects on measurement error across surveys and over time

**Fig. 2:**
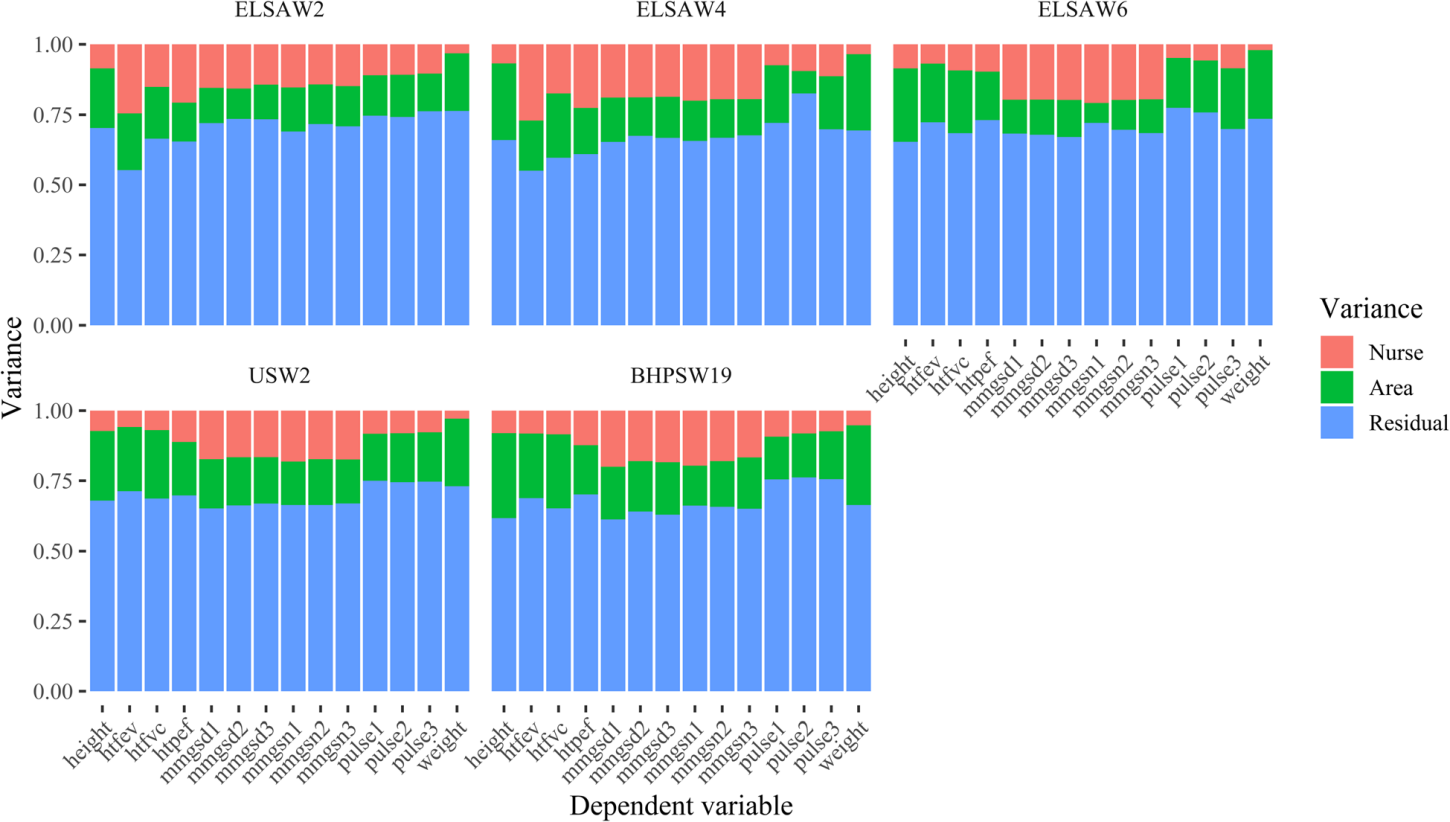
Variance decomposition for nurse visit measures by survey and wave after controlling for areas and respondent characteristics

Next, we investigate if these nurse effects vary over time and data source. Figure 2 disentangles the variation by biomeasure, survey, and wave. Only small variations are observed: the average ICC in waves 2, 4, and 6 of ELSA are 13.9, 15.8, and 12.5%, respectively, whereas the average ICCs for the US wave 2 and BHPS wave 19 are 11.5 and 12.7%, respectively. For ELSA, it appears that the effects of nurses on lung capacity measures are higher in waves 2 to 4 compared to wave 6. One explanation for this difference is the change in the model of spirometer used in ELSA wave 6 [48]. On the other hand, waves 4 and 6 show higher levels of nurse effects on grip-strength compared to wave 2, suggesting that nurse effects are worsening over time. For UKHLS the effects are more consistent although the average nurse effect is also higher for BHPS19 than for USW2.

Comparing ELSAW2 with USW2 we see that nurse effects on lung capacity are higher in the former while the effect on grip strength is higher in the latter. On average, nurse effects are larger in ELSA (around 14%) than in the US and BHPS (around 12%).

**Fig. 3:**
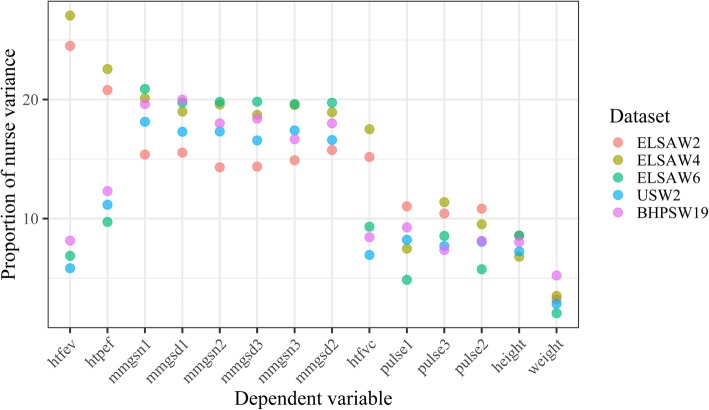
Proportion of nurse variance for biomeasures by survey and wave after controlling for respondent characteristics

Another way to visualize the differences in nurse effects over time and between data sources is shown in Fig. 3. Here, the higher levels of nurse effects for lung capacity in waves 2 and 4 of ELSA are more obvious. One can also see lower nurse effects on grip strength in ELSAW2 compared to the other data sources. Further, the figure confirms the low levels of nurse effects on the measures of pulse, height and weight in all data sources.

### The impact of nurse characteristics on measurement error effects

Lastly, we investigate to what degree nurse characteristics (nurse experience and nurse age) explain the nurse measurement error effects found above. Overall, nurse characteristics explain only a very small proportion of variation. The average proportion of variation explained with nurse characteristics for each biomeasure ranges from a minimum of 0.1% to a maximum of 1.8%. Thus, it appears that stronger background data are needed in order to understand the mechanisms that lead to nurse measurement effects and how to tackle them.

## Discussion

This study aimed to investigate whether nurses influence the measurement of anthropometric measures as well as physical performance measures in large, nationally-representative biosocial surveys. The investigation concentrated on 14 biomeasures collected in two surveys over five waves of data collection. Nurse effects were estimated using a cross-classified multilevel model that separated area and nurse effects and controlled for respondent characteristics. Overall, we found a medium-sized nurse effect. Across all biomeasures and data sources, nurses explained about 13% of the observed variation, on average. This was shown to vary significantly from close to 0 % for some measures, such as pulse, height and weight and up to 27% for grip strength measurements. The finding that physical performance measures are more susceptible to nurse effects compared to anthropometric measures is consistent with other nurse-led studies, which have found measures of height and weight to be least prone to measurement error compared to other measures which require more careful administration and use of more sophisticated equipment [28].

Further, we investigated to what degree nurse effects vary by time and survey. This issue is important as varying nurse effects can distort estimates of change and comparisons between studies. Some differences over time and study were observed, but they did not reveal a consistent pattern. Nurse effects were larger for measures of lung capacity in ELSA compared to US and BHPS. On the other hand, nurse effects on grip strength were higher in US and BHPS compared to ELSA. Looking at comparisons over time it was found that ELSA wave 6 had smaller nurse effects on lung capacity compared to earlier waves 2 and 4, suggesting that nurse measurement improves over the course of the study for this particular measure. This pattern, however, was reversed for grip strength where the largest nurse effects were observed in the later waves of the study.

Finally, we attempted to explain these nurse effects using two important nurse characteristics measured in all five datasets: nurse experience and nurse age. Overall, the models explained only a very small proportion of the nurse effects (less than 2%) when the nurse characteristics were introduced. It is clear that additional nurse characteristics are needed to better understand the processes that help explain measurement error effects.

This study has two important limitations. Firstly, in the absence of random allocation of nurses to respondents a modeling approach to control for confounding of nurse and area effects and respondent characteristics was used. Although this approach makes the assumption that both effects can be separated through control variables, it does yield good variation and highlights differences between biomeasures and data sources in nurse effects. Further, this is a standard approach used in investigating observer effects in observational studies [49]. Secondly, the analysis of nurse characteristics that explain nurse measurement effects is based on only two variables: experience and age. Although these variables are important from a theoretical viewpoint, we found that they have poor explanatory ability. Thus, more information about the nurses is needed to understand the processes underlying nurse measurement effects and allow researchers to control for these effects.

From these results, it is clear that making available the nurse ID variable as well as nurse characteristics can be useful not only for modelling nonresponse but also for estimating and accounting for nurse effects on measurement error. The results also suggest the need to perform sensitivity analyses that take into account the nurse effects on measurement when using data collected by nurses. These effects are similar to those found in the interviewer effects literature and highlight that objective measures of health collected by nurses are not impervious to measurement error.

Lastly, more research is needed to understand the mechanisms of nurse measurement error effects. This can be done either through qualitative research or by collecting more detailed information about nurse attitudes and behaviors, similar to what has been done in the lay interviewer effects literature [19]. This can, in turn, inform procedures or training programs that can help minimize this unnecessary source of measurement error.

This research is among the first to investigate the effects of nurses on biological data collection in a population-representative household sample survey. This was informed by two distinct literatures: the medical research in biological data collection and the survey methodology research on interviewer effects. Our results indicate that this has the potential to be an important research area and opens up some intriguing research questions. For example, do these finding generalize to other types of health care professionals? Are these differences in nurse effects by type of measurement consistent across contexts and survey institutes? How do these effects influence substantive analyses? We hope this study will trigger avenues for research that can answer such questions.

## Conclusions

Our study showed that some objective health measures collected by nurses in household surveys are susceptible to non-trivial amounts (up to 27%) of measurement error variability at the nurse level. Physical performance measures tended to be more influenced by nurse measurement error than anthropometric measures. Nurse characteristics did not explain a significant amount of the observed measurement error. We recommend that researchers account for this unnecessary source of error by incorporating nurse ID numbers in their analysis, or performing a sensitivity analysis to determine whether substantive findings are affected by nurse measurement effects. Moreover, a richer array of nurse characteristics should be included in biosocial data releases to further allow researchers to control for this.

## Supplementary information

**Additional file 1.** Descriptive statistics of dependent variables

## Data Availability

Data from Understanding Society (US) and the English Longitudinal Study of Ageing (ELSA) are available from the UK Data Service for researchers who meet the criteria for access to confidential data, under conditions of the End User License http://ukdataservice.ac.uk/media/455131/cd137-enduserlicence.pdf. The Understanding Society data can be accessed from: https://beta.ukdataservice.ac.uk/datacatalogue/series/series?id=2000053. The English Longitudinal Study of Ageing data can be accessed from: http://discover.ukdataservice.ac.uk/series/?sn=200011. Contact with the UK data service regarding access to Understanding Society and the English Longitudinal Study of Ageing can be made through the website http://ukdataservice.ac.uk/help/get-in-touch.aspx, by phone + 44 (0)1206 872143, or by email at help@ukdataservice.ac.uk.

## Abbreviations

BHPS: British Household Panel Survey
BHPSW19: British Household Panel Survey, Wave 19
CM: Centimeters
ELSA: English Longitudinal Study of aAgeing
ELSAW2: English Longitudinal Study of Ageing, Wave 2
ELSAW4: English Longitudinal Study of Ageing, Wave 4
ELSAW6: English Longitudinal Study of Ageing, Wave 6
HTFEV: Lung capacity amount of air that can be blown out in 1 sec
HTFVC: Lung capacity amount of air that can be forcibly blown out after a full inhalation
HTPEV: Lung capacity speed of air moving out of lungs
ICC: Intraclass Correlation Coefficient
KG: Kilograms
MMGSD: Grip strength for dominant hand
MMGSN: Grip strength for non-dominant hand
UKHLS: UK Household Longitudinal Study
US: Understanding Society – UK Household Longitudinal Study
USW2: Understanding Society – UK Household Longitudinal Study, Wave 2

## Footnotes

1 See http://mc-stan.org/rstanarm/articles/priors.html and http://mc-stan.org/rstanarm/articles/glmer.html
