# Supplementary material for: Nurse effects on measurement error in household biosocial surveys
Source: BMC Med Res Methodol. 2020 Feb 27;20:45. doi: 10.1186/s12874-020-00922-2 (PMC7047401; doi:10.1186/s12874-020-00922-2)
Supplement: Supplementary file 1 — Additional file 1. Descriptive statistics of dependent variables [file 12874_2020_922_MOESM1_ESM.docx]

**APPENDIX**

**Descriptive statistics of dependent variables**

| **Variable** | **Statistic** | **ELSAW2** | **ELSAW4** | **ELSAW6** | **USW2** | **BHPSW19** |
| --- | --- | --- | --- | --- | --- | --- |
| **height** | mean | 165.3 | 165.76 | 165.76 | 167.35 | 167.2 |
|  | SD | 9.53 | 9.61 | 9.49 | 9.61 | 9.69 |
| **htfev** | mean | 2.31 | 2.41 | 2.38 | 2.91 | 2.95 |
|  | SD | 0.85 | 0.85 | 0.82 | 0.91 | 0.94 |
| **htfvc** | mean | 3.16 | 3.27 | 3.29 | 3.82 | 3.87 |
|  | SD | 1.07 | 1.08 | 1.08 | 1.09 | 1.13 |
| **htpef** | mean | 369.45 | 376.58 | 6.59 | 7.42 | 7.51 |
|  | SD | 143.38 | 146.41 | 2.39 | 2.31 | 2.34 |
| **mmgsd1** | mean | 28.98 | 29.06 | 28.21 | 31.33 | 31.6 |
|  | SD | 11.43 | 11.5 | 11.03 | 11.68 | 11.92 |
| **mmgsd2** | mean | 29.4 | 29.41 | 28.7 | 31.61 | 31.88 |
|  | SD | 11.65 | 11.7 | 11.2 | 11.76 | 11.97 |
| **mmgsd3** | mean | 29.58 | 29.64 | 28.9 | 31.85 | 32.14 |
|  | SD | 11.79 | 11.89 | 11.32 | 11.85 | 12.01 |
| **mmgsn1** | mean | 26.28 | 26.5 | 25.93 | 28.59 | 28.69 |
|  | SD | 10.58 | 10.54 | 10.17 | 10.77 | 11.15 |
| **mmgsn2** | mean | 27.03 | 27.12 | 26.41 | 29.21 | 29.41 |
|  | SD | 11.01 | 11.02 | 10.57 | 11.09 | 11.28 |
| **mmgsn3** | mean | 27.26 | 27.33 | 26.57 | 29.45 | 29.67 |
|  | SD | 11.12 | 11.18 | 10.72 | 11.22 | 11.36 |
| **pulse1** | mean | 68 | 75.5 | 72.93 | 69.37 | 69.23 |
|  | SD | 11.77 | 86.61 | 77.13 | 11.27 | 11.5 |
| **pulse2** | mean | 67.95 | 79.78 | 77.81 | 69.61 | 69.41 |
|  | SD | 11.55 | 106.47 | 101.43 | 11.18 | 11.35 |
| **pulse3** | mean | 68.07 | 85.32 | 82.13 | 69.97 | 69.86 |
|  | SD | 11.39 | 127.39 | 118.5 | 11.14 | 11.27 |
| **weight** | mean | 76.48 | 77.66 | 77.68 | 78.05 | 78.47 |
|  | SD | 15.35 | 15.89 | 15.83 | 16.43 | 16.81 |

**Descriptive statistics for independent variables in ELSA waves 2, 4 and 6**

|  |  | **ELSAW2** | | **ELSAW4** | | **ELSAW6** | |
| --- | --- | --- | --- | --- | --- | --- | --- |
| **Variable** | **Code** | **Freq.** | **Perc.** | **Freq.** | **Perc.** | **Freq.** | **Perc.** |
|  |  |  |  |  |  |  |  |
| **Sample** |  | 7554 | 100 | 8436 | 100 | 7880 | 100 |
|  |  |  |  |  |  |  |  |
| **Female** |  | 4145 | 54.9 | 4627 | 54.8 | 4336 | 55 |
| **White** |  | 7415 | 98.2 | 8212 | 97.3 | 7636 | 96.9 |
| **Age** | (48,65] | 3839 | 50.8 | 4632 | 54.9 | 3799 | 48.2 |
|  | (65,75] | 2279 | 30.2 | 2444 | 29 | 2497 | 31.7 |
|  | (75,90] | 1436 | 19 | 1360 | 16.1 | 1584 | 20.1 |
| **Has partner** |  | 5230 | 69.2 | 6035 | 71.5 | 5539 | 70.3 |
| **Owns home** |  | 6296 | 83.3 | 7127 | 84.5 | 6659 | 84.5 |
| **Lives alone** |  | 3041 | 40.3 | 4004 | 47.5 | 4023 | 51.1 |
| **Education** | Degree | 934 | 12.4 | 1462 | 17.3 | 1434 | 18.2 |
|  | A level | 1435 | 19 | 1903 | 22.6 | 1798 | 22.8 |
|  | O level | 2342 | 31 | 2761 | 32.7 | 2767 | 35.1 |
|  | No qual. | 2843 | 37.6 | 2310 | 27.4 | 1881 | 23.9 |
| **General health** | Poor | 526 | 7 | 588 | 7 | 596 | 7.6 |
|  | Fair | 1511 | 20 | 1562 | 18.5 | 1521 | 19.3 |
|  | Good | 2422 | 32.1 | 2698 | 32 | 2501 | 31.7 |
|  | Very good | 2146 | 28.4 | 2494 | 29.6 | 2339 | 29.7 |
|  | Excellent | 949 | 12.6 | 1094 | 13 | 923 | 11.7 |
| **Long ill** |  | 5412 | 71.6 | 6214 | 73.7 | 5884 | 74.7 |
| **London** |  | 625 | 8.3 | 704 | 8.3 | 652 | 8.3 |
| **North** |  | 2281 | 30.2 | 2353 | 27.9 | 2198 | 27.9 |
| **Nurse age** | 35-54 | 1299 | 17.2 | 2115 | 25.1 | 2004 | 25.4 |
|  | 55-64 | 2501 | 33.1 | 2421 | 28.7 | 3367 | 42.7 |
|  | Over 64 | 3754 | 49.7 | 3900 | 46.2 | 2509 | 31.8 |
| **Nurse experience** | 1-5 years | 1673 | 22.1 | 1293 | 15.3 | 1852 | 23.5 |
|  | 6-10 years | 1624 | 21.5 | 3234 | 38.3 | 3051 | 38.7 |
|  | 11-15 years | 2181 | 28.9 | 2039 | 24.2 | 1607 | 20.4 |
|  | 16 years or more | 2076 | 27.5 | 1870 | 22.2 | 1370 | 17.4 |

**Descriptive statistics for independent variables in Understanding Society wave 2 and BHPS wave 19**

|  |  | **USW2** | | **BHPSW19** | |
| --- | --- | --- | --- | --- | --- |
| **Variable** | **Code** | **Freq.** | **Perc.** | **Freq.** | **Perc.** |
|  |  |  |  |  |  |
| **Sample size** |  | 15346 | 100 | 4774 | 100 |
|  |  |  |  |  |  |
| **Female** |  | 8642 | 56.3 | 2661 | 55.7 |
| **Age** | (16,35] | 3287 | 21.4 | 1052 | 22 |
|  | (35,55] | 5686 | 37.1 | 1772 | 37.1 |
|  | (55,75] | 5064 | 33 | 1460 | 30.6 |
|  | (75,102] | 1309 | 8.5 | 490 | 10.3 |
| **Has partner** |  | 10258 | 66.8 | 3996 | 83.7 |
| **Owns home** |  | 11291 | 73.6 | 3712 | 77.8 |
| **Urban** |  | 11724 | 76.4 | 1475 | 30.9 |
| **HH size** | 1 | 2703 | 17.6 | 786 | 16.5 |
|  | 2 | 6063 | 39.5 | 1820 | 38.1 |
|  | 3 | 2666 | 17.4 | 905 | 19 |
|  | 4 | 2558 | 16.7 | 881 | 18.5 |
|  | 5 | 1002 | 6.5 | 270 | 5.7 |
|  | 6 | 260 | 1.7 | 70 | 1.5 |
|  | 7 | 64 | 0.4 | 24 | 0.5 |
|  | 8 | 26 | 0.2 | 5 | 0.1 |
|  | 9 | 2 | 0 | 8 | 0.2 |
|  | 10 | 2 | 0 | 5 | 0.1 |
| **Education** | Higher | 5414 | 35.3 | 1441 | 30.2 |
|  | A level | 2808 | 18.3 | 1099 | 23 |
|  | GCSE | 3045 | 19.8 | 1099 | 23 |
|  | Other/no qual | 4079 | 26.6 | 1135 | 23.8 |
| **Interest in voting** |  | 1749 | 11.4 | 460 | 9.6 |
| **London** |  | 1239 | 8.1 | 218 | 4.6 |
| **North** |  | 8273 | 53.9 | 2781 | 58.3 |
| **General health** | Poor | 1042 | 6.8 | 321 | 6.7 |
|  | Fair | 2433 | 15.9 | 784 | 16.4 |
|  | Good | 4342 | 28.3 | 1332 | 27.9 |
|  | Very good | 5221 | 34 | 1627 | 34.1 |
|  | Excellent | 2308 | 15 | 710 | 14.9 |
| **Long ill** |  | 6112 | 39.8 | 1849 | 38.7 |
| **Nurse experience** | 1-5 years | 8809 | 57.4 | 2911 | 61 |
|  | 6-10 years | 3734 | 24.3 | 934 | 19.6 |
|  | 11-15 years | 1661 | 10.8 | 511 | 10.7 |
|  | 16 years or more | 1086 | 7.1 | 337 | 7.1 |
|  | Missing | 56 | 0.4 | 81 | 1.7 |
| **Nurse age** | (0,40] | 822 | 5.4 |  |  |
|  | (40,50] | 3865 | 25.2 |  |  |
|  | (50,60] | 6253 | 40.7 |  |  |
|  | over 60 | 4350 | 28.3 |  |  |
|  | Missing | 56 | 0.4 |  |  |
| **Nurse age** | (0,54] |  |  | 1974 | 41.3 |
|  | (54,64] |  |  | 1614 | 33.8 |
|  | over 64 |  |  | 1105 | 23.1 |
|  | Missing |  |  | 81 | 1.7 |
